# Experimental and theoretical assessment to investigate the impact of Gast Reg drug on the copper corrosion control in an acidic environment

**DOI:** 10.1038/s41598-024-84041-8

**Published:** 2025-02-12

**Authors:** S. M. Syam, H. Nady, Salah Eid, Emad E. El-Katori

**Affiliations:** 1 Chemistry department, Faculty of science, Benha University, Benha, Egypt; 2 Department of Chemistry, College of Science, Jouf University, 72341 Sakaka, Aljouf Saudi Arabia; 3 Department of Chemistry, Faculty of Science, New Valley University, El-Kharga, 72511 Egypt

**Keywords:** Gast Reg drug, Adsorption, Copper, Acid corrosion, Impedance, Monte Carlo simulations, Corrosion, Physical chemistry

## Abstract

Herein, a corrosion inhibitor called the Gast Reg drug (**GRD**) was used to prevent the copper from corroding. The aggressive solution used in this investigation was HCl acid solution. A mix of electrochemical and quantum investigations are used to assess **GRD**’s anti-corrosion properties. It has been discovered that the **GRD** is essential for stopping copper from corroding in a 2 M HCl solution. The study’s results indicated that **GRD** shown considerable corrosion prevention capabilities for copper in 2 M HCl solution. The inhibitory effectiveness of **GRD** was seen to rise with higher concentrations of **GRD**. It is noteworthy that the maximum levels of inhibitory effectiveness (82.1%) for HCl solution were obtained at 123.87 × 10^− 5^ M. The primary cause of GRD’s anti-corrosion properties is its propensity to adsorb on the surface of copper via its heteroatoms. The inhibitor’s adsorption behavior was described using the Langmuir model. Surface assessments with Energy Dispersive X-ray (EDX), Scanning Electron Microscope (SEM), and Atomic Force Microscope (AFM) demonstrated the development of a prominent adsorbed film on the copper surface. The correlation between molecule structure and its inhibitory effect has been investigated and analyzed using DFT and Monte Carlo simulation. The actual adsorption occurs through a variety of active centers and physical and chemical processes that are coordinated with the calculated quantum parameters. The outcomes gathered from electrochemical, surface, and theoretical studies are well correlated.

## Introduction

Copper and copper-based alloys are highly sought after in certain applications because of their exceptional resistance to corrosion, good physical qualities, workability, and advantageous thermal and electrical conductivities^1^. They can be employed in heat exchangers, water transport systems, water treatment units, saltwater condensers, tubes, valves, and fittings, as well as deployment in chemical, petrochemical industries, and electricity generation and desalination plants^2–5^. Despite the numerous utilities of copper, the challenge of mitigating its susceptibility to corrosion remains paramount. An established and cost-effective approach to address this challenge involves the application of corrosion inhibitors, with a particular emphasis on organic inhibitors^6,7^. Organic inhibitors typically function by adhering to the metal surface, thereby creating protective barriers that impede the corrosive action of the surrounding environment and attenuate metal degradation^8,9^. Contemporary research has extended the repertoire of corrosion inhibitors to include medicinal compounds employed in aqueous solutions^10,11^. Notably, pharmaceuticals, characterized by structural features such as aromatic rings and heteroatom active sites conducive to facile adsorption, emerge as promising candidates for corrosion inhibition in metal systems^12^. Extensive investigation into the corrosion inhibitory potential of various drugs has unfolded^13–16^, with their blocking effect due to attaching to the metal surface and stopping further breakdown.

This study aims to examine the efficacy of the Gast Reg drug (**GRD**) in mitigating the corrosion of copper electrodes immersed in 2 M HCl. Employing a diverse suite of electrochemical measurements, including open-circuit potential (OCP), potentiodynamic polarization (PP), and electrochemical impedance spectroscopy (EIS), along with surface studies using SEM/EDX and AFM, this gives a full picture of how the rust is stopped. By using equivalent circuit models, actual impedance data may be accurately matched with theoretical values. This enables a detailed comprehension of the electrochemical behavior occurring at the interface between a metal and a solution across varying conditions.

The inhibitor’s adsorption free energy on the copper surface is also calculated, revealing the chemical interaction between the inhibitor and the copper surface. DFT computations and Monte Carlo (MC) simulations are used to explore the inhibitor’s molecular structure and protective properties. This multifaceted approach aims to unravel the intricate interplay between the Gast Reg drug and copper surfaces, offering significant observations into the development of effective methods for preventing corrosion.

## Materials and methods

### The tested solutions

The prepared solutions for experimentation were meticulously crafted using analytical-grade chemical reagents and distilled water. Introducing a novel inhibitor, the Gast Reg drug (**GRD**), further enhanced the scope of the study. Manufactured by the Medical Union Pharmaceuticals in Abu-Sultan, Ismailia, Egypt, under license from Newport Pharmaceuticals Limited in Dublin, Ireland, Gast Reg is a pharmaceutical compound containing 24 mg of **Trimebutine** per 5 ml in a total volume of 125 ml. The required volumes to achieve the desired inhibitor concentrations were meticulously determined and subsequently prepared through dilution processes.

**Fig. 1:**
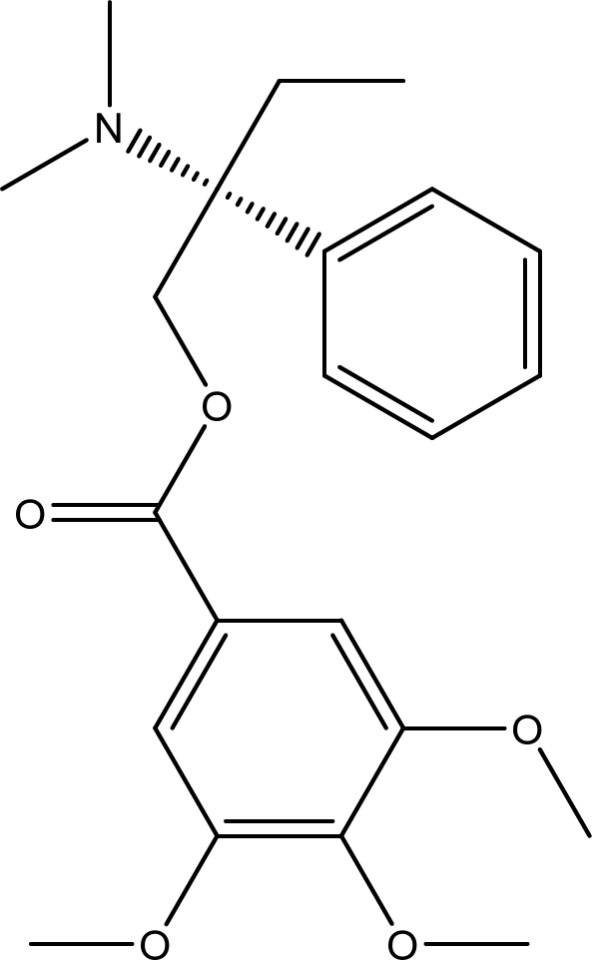
The chemical structures of Trimebutine.

**Trimebutine**, identified chemically as 2-(Dimethylamino)-2-phenylbutyl 3,4,5-trimethoxybenzoate, possesses a molar mass of 387.5 g/mol, and its chemical formula is denoted as C_22_H_29_NO_5_. Figure 1 illustrates the structural representation of **Trimebutine**.

### Preparation of the electrode

The working electrode, derived from a copper plate, was meticulously fashioned by securing it within glass tubing possessing a suitable internal diameter. This secure fixation was achieved through the application of chemically resistant epoxy resin, ensuring a robust bond. The resultant electrode configuration exhibited a front surface area of 0.12 cm², which was designated for direct contact with the corrosive solutions under investigation. A prerequisite to each experimental iteration involved a systematic preparatory procedure for the copper electrode surface. This involved successive abrasion using 1200/2000/2500 grit emery papers, tightly wrapped around a smooth polishing cloth. Subsequently, the electrode underwent thorough washing with tri-distilled water. Post-cleansing, the electrode was promptly dipped in the designated corrosive solutions.

### Electrochemical cell

The electrochemical cell used in this study was a three-electrode configuration, consisting of an all-glass setup. The reference electrode employed was a saturated calomel electrode (SCE), while a platinum wire counter electrode was utilized for electrical connectivity. The electrochemical measurements were conducted within a 2 M hydrochloric acid solution, both in the absence and presence of diverse concentrations of the corrosion inhibitor (**GRD**). All experiments transpired at a controlled temperature of 25 °C, in stagnant 2 M HCl solutions. Each experimental iteration utilized a freshly prepared solution to ensure methodological rigor and consistency.

### Electrochemical measurements

The copper electrode’s open circuit potential was measured individually against the saturated calomel electrode (SCE) reference electrode (E_o_ = 244 mV compared with the standard hydrogen electrode, SHE). After that, the Versa STAT 4 equipment and the Versa Studio electrochemistry software program were used to do potentiodynamic polarization (PDP) and electrochemical impedance spectroscopy (EIS) experiments, maintaining constant experimental conditions. For the potentiodynamic polarization experiments, a quasi-stationary state was achieved with a scan rate of 5 × 10^− 3^ V s^− 1^. The total impedance (Z) and phase shift plots were acquired at the Open Circuit Potential (OCP) within a frequency range from 10^5^ to 10^− 1^ Hz, employing a sine wave of 10 mV amplitude.

### Surface examination

Copper coupons were abrasively treated with different emery sheets up to a 2500 grade, and then immersed in 2 M HCl solutions, both with and without the presence of 123.87 × 10^− 5^ M of Gast Reg drug (GRD), for a duration of 24 h. After this exposure period, the samples were subjected to drying and, subsequently, were placed within a desiccator after being cleansed with distilled water. The surface examination was examined via scanning electron microscopy (SEM) analysis utilizing the BED-C 10.0VKV instrument manufactured by Jeol. This SEM apparatus was augmented with an energy-dispersive X-ray (EDX) unit, facilitating a comprehensive analysis of the surface composition.

### DFT computations and MC simulations

Utilizing Accelrys Materials Studio 7.0, theoretical investigations were conducted utilizing the DMol3 module for Density Functional Theory (DFT) calculations and the adsorption locator module for Monte Carlo simulations. The geometric optimization of the inhibitor Gast Reg drug (GRD) was conducted in DFT calculations using the B3LYP method with a DNP basis set and COSMO solvation conditions^17^. The Forcite module was first used to optimize the structure of the inhibitor for Monte Carlo simulations. After Monte Carlo searches, the adsorption finder module found the inhibitor’s optimal adsorption configurations on the Cu (1 1 1) surface, allowing an evaluation of the inhibitor molecules’ protective efficiency^18^. The inhibitor’s adsorption, water molecules, and the copper (111) interface were all included in the simulation box, delineated with dimensions of 25.56 Å × 25.56 Å × 38.78 Å, utilizing the COMPASS force field^19^. It is pertinent to note that all specifics and inputs pertinent to the theoretical calculations have been comprehensively detailed in prior publications^17,18^, ensuring transparency and reproducibility of the computational methodologies employed in this study.

## Results and discussion

### OCP measurements

**Fig. 2:**
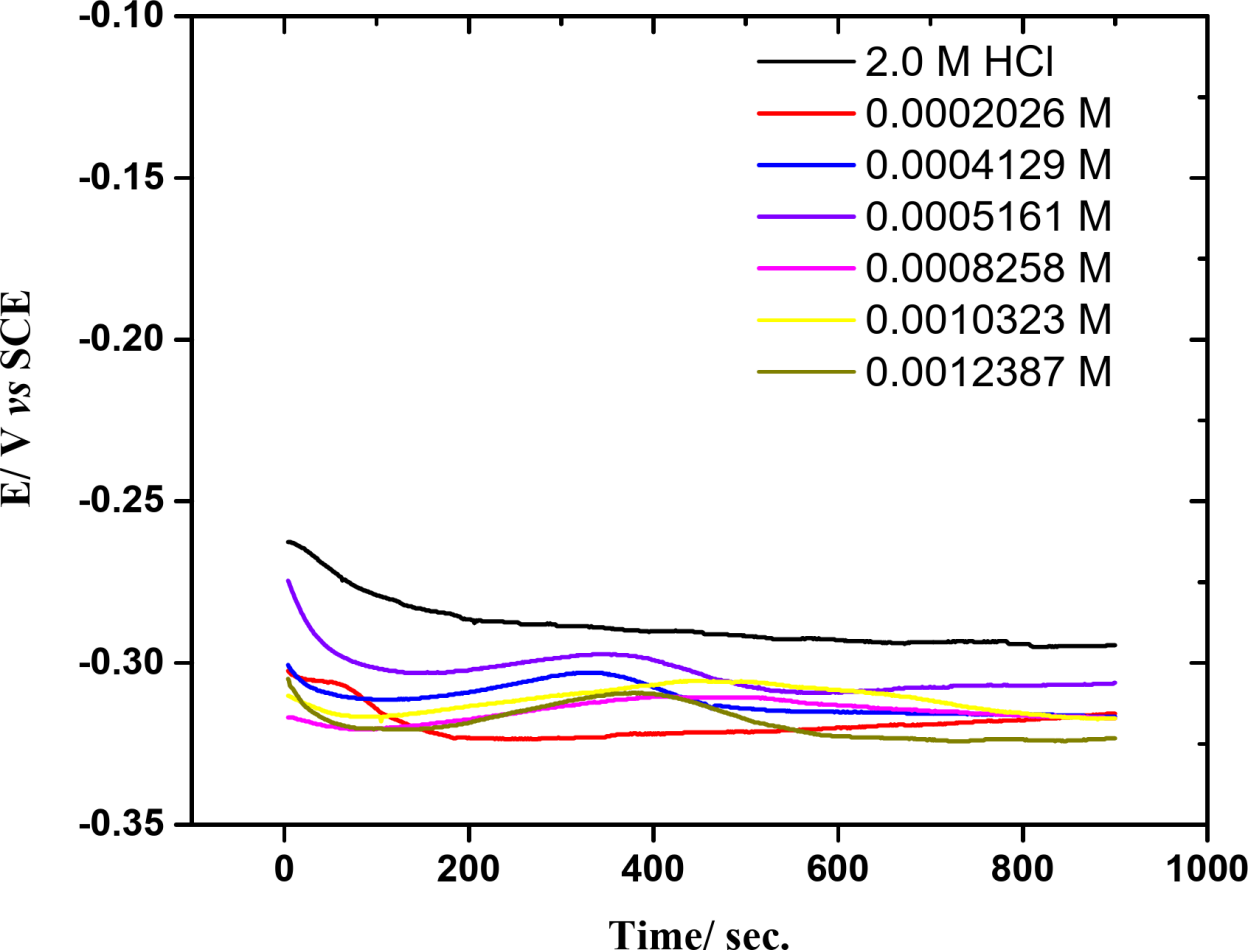
Time-potential curves for copper in 2 M HCl solution without and with various concentrations of **GRD** at 25 ± 1 °C.

OCP curves were constructed by measuring the copper electrode potential relative to the SCE. As shown in Fig. 2, the Cu electrode’s OCP was measured after electrode dipped in a 2 M HCl solution with and without varying dosages of **GRD** to achieve a steady state. Every measurement was done at a temperature of 25 °C. The presence of **GRD** in 2 M HCl shifts *E*_ss_ to negative potentials compared to the *E*_ss_ of the copper electrode in inhibitor - free acid solution. The *E*_ss_ shift may reflect that the heteroatoms (N & O) present on **GRD** compound are binding to active corrosion sites, slowing copper breakdown^20–22^.

### PDP measurements

**Fig. 3:**
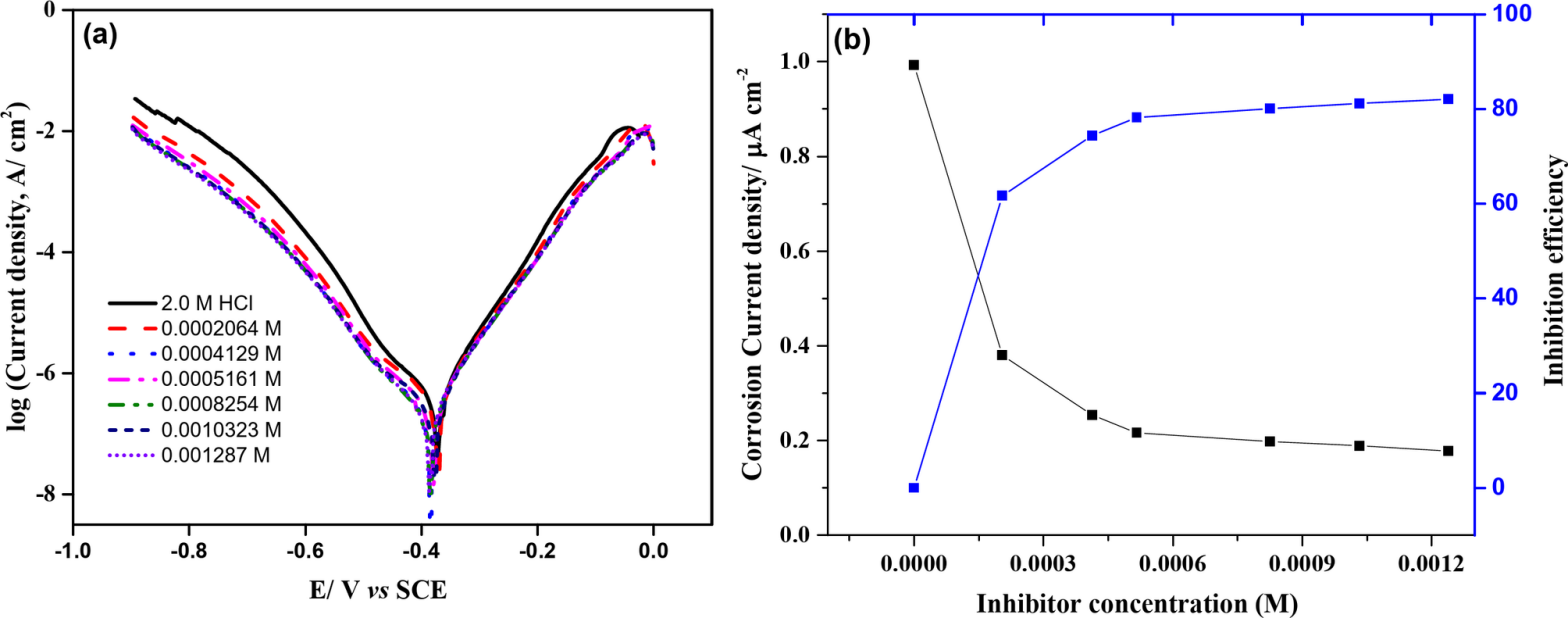
(**a**) Potentiodynamic polarization curves for the corrosion of copper in 2 M HCl in the absence and presence of various concentrations of **GRD** at 25 ± 1 °C, (**b**) Plot of inhibition efficiency & corrosion current density versus inhibitor concentration.

Potentiodynamic polarization studies of the Cu electrode were performed in 2 M HCl medium, both with and without different Gast Reg drug (**GRD**) doses. Figure 3 illustrates a representative presentation demonstrating the impact of **GRD** concentrations on the polarization curve of a copper electrode. Various corrosion parameters, including the corrosion potential (*E*_*corr*_), corrosion current density (*i*_*corr*_), and the anodic (*β*_*a*_) and cathodic (*β*_*c*_) Tafel slopes, were calculated from distinct polarization curves. These values are tabulated in Table 1 for solutions without and with **GRD**. A discernible and consistent trend emerges, as *i*_*corr*_ exhibits a marked reduction in the presence of **GRD**. The value of corrosion potential (*E*_*corr*_) slightly affected in the presence of the inhibitor, which suggests a mixed adsorption process. Figure 3a further illustrates that while the presence of **GRD** doesn’t alter the general features of the polarization curves, it significantly diminishes the corrosion current density, indicative of a lowered corrosion rate^23^. This suggests that **GRD** molecules do not modify the underlying mechanism of the corrosion process but effectively reduce both cathodic and anodic current densities, affirming the role of **GRD** as a mixed inhibitor. Measured current densities in the presence (*i*_*corr*_(inh)) and absence (*i*_*corr*_) of **GRD** were used to quantify corrosion inhibition efficiency (*η*), according to the formula^24^: *η* = [($\:\frac{icorr-icorr\:\left(inh\right)}{icorr}$)× 100]. The estimated inhibition efficiencies, reported in Table 1, underscore the robust corrosion inhibition properties of **GRD**. The anodic dissolving process is hindered, and the cathodic reaction is retarded when **GRD** is added to the corrosive environment. A minimal concentration is sufficient to achieve a significant level of inhibition. One possible reason for this observation is that a small number of inhibitor molecules were able to cover a large area on the metal substrate by attaching to it horizontally. The figure demonstrates that, at this juncture, the inhibition efficiency value is mostly unaffected by the inhibitor concentration. After the surface becomes nearly saturated with adsorbed molecules, it is plausible to conclude that the inhibition efficacy remains unchanged with increasing inhibitor concentration^25^. Furthermore, noticeable alterations in cathodic Tafel slope values suggest the prevalence of the cathodic process over the anodic one^26^.

**Table 1:** Polarization parameters of copper dissolution in stagnant 2 M HCl in the absence and presence of different concentrations of inhibitor **GRD**.

Conc., M	E_corr_/mV_(SCE)_	i_corr_/µA cm^− 2^	β_a_/mV dec^− 1^	β_c_/mV dec^− 1^	Θ	η%
0	− 356.0	0.993	84.6	-149.9	-	0
0.0002064	− 364.4	0.380	65.9	-111.8	0.617	61.7
0.0004129	− 393.1	0.254	83.1	-111.7	0.744	74.4
0.0005161	− 386.0	0.216	72.3	-98.8	0.782	78.2
0.0008258	− 389.9	0.198	71.2	-101.8	0.801	80.1
0.0010323	− 390.8	0.189	73.2	-88.1	0.812	81.2
0.0012387	− 394.7	0.178	72.3	-87.1	0.821	82.1

### Electrochemical impedance spectroscopic measurements, EIS

**Fig. 4:**
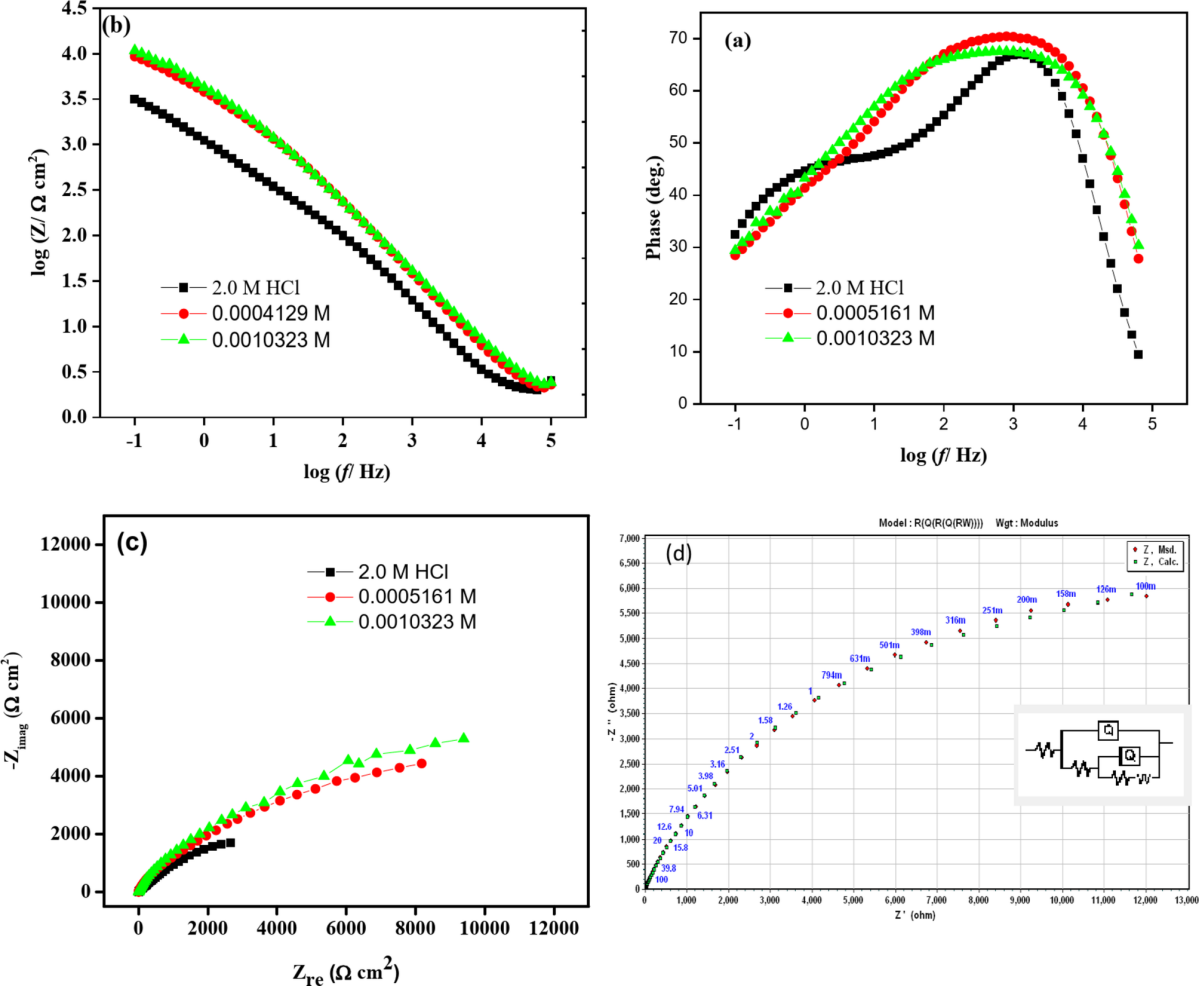
Electrochemical impedance Spectrum investigation: (**a** &** b**) Bode and (**c**) Nyquist curves of copper dissolution in 2 M HCl in the absence and presence of various concentrations of **GRD** at 25 ± 1 °C, (**d**) Electrical equivalent model for simulation of the impedance spectra.

Electrochemical Impedance Spectroscopy (EIS) stands as a formidable methodology for assessing corrosion rates, particularly in the context of coatings, passive films, and adsorbed layers. A notable advantage of EIS lies in its utilization of a minute alternating current signal, which minimally disrupts the electrode surface morphology, or the characteristics being measured. Furthermore, the approach has the possibility of simulating experimental impedance outputs utilizing pure electrical models. This process facilitates the deduction of numerical values that correspond to the pertinent physical and/or chemical characteristics of the electrochemical system and helps to validate or refute mechanistic models^27–32^. The copper electrode’s impedance data in 2 M HCl solutions with and without Gast Reg drug (**GRD**) concentrations is shown as Nyquist and Bode curves in Fig. 4 and summarized in Table 2. The Bode curves (Fig. 4a and b) prominently exhibit a two-phase maximum, with an increase in the phase angle and broadening of the phase maximum correlating with higher inhibitor concentrations, indicative of a concurrent reduction in the corrosion rate of copper. The Nyquist curve (Fig. 4c) manifests a characteristic profile featuring a depressed two capacitive loop at high and low frequencies, along with two semicircles. This behavior is attributed to a dual-time constant, encompassing relaxation effects related to adsorption phenomena. It signifies that the corrosion process is primarily under the influence of charge transfer control^20,24,27^. The high-frequency semicircle is usually linked with double-layer capacitance relaxation, with its diameter signifying charge-transfer resistance^30,31^. The second loop at low-to-intermediate frequency considers the formation of an adsorption inhibitor layer, and the resistance increases with increasing the inhibitory concentrations^20^. In the analysis of impedance data, the provided software associated with the impedance system was employed, utilizing a dispersion formula. As shown in the inset of Fig. 4d, utilizing a fundamental equivalent circuit model, the experimental impedance measurements were matched to the theoretical data. This comprehensive electrochemical analysis provides valuable insights into the corrosion processes and the efficacy of the inhibitor, **GRD**, in altering the charge transfer dynamics at the copper electrode interface.

**Table 2:** Electrochemical kinetic parameters obtained from EIS for copper in stagnant 2 M HCl in the absence and presence of different concentrations of inhibitor **GRD**.

Conc. M	*R*_s_ (Ω cm^2^)	*R*_1_ (Ω cm^2^)	Q_1_ Y_o_ (Ω^−1^ cm^− 2^ s^n2^)	Rad (Ω cm^2^)	Q_2_ Y_o_ (Ω^−1^ cm^− 2^ s^n2^)
0	2.0	0.01	7.2 × 10^− 6^	177	16.7 × 10^− 5^
0.0005161	2.15	19.2	2.7 × 10^− 6^	6959	4.6 × 10^− 5^
0.0010323	2.29	16.15	2.2 × 10^− 6^	9375	4.4 × 10^− 5^

### SEM/EDX and AFM studies

**Fig. 5:**
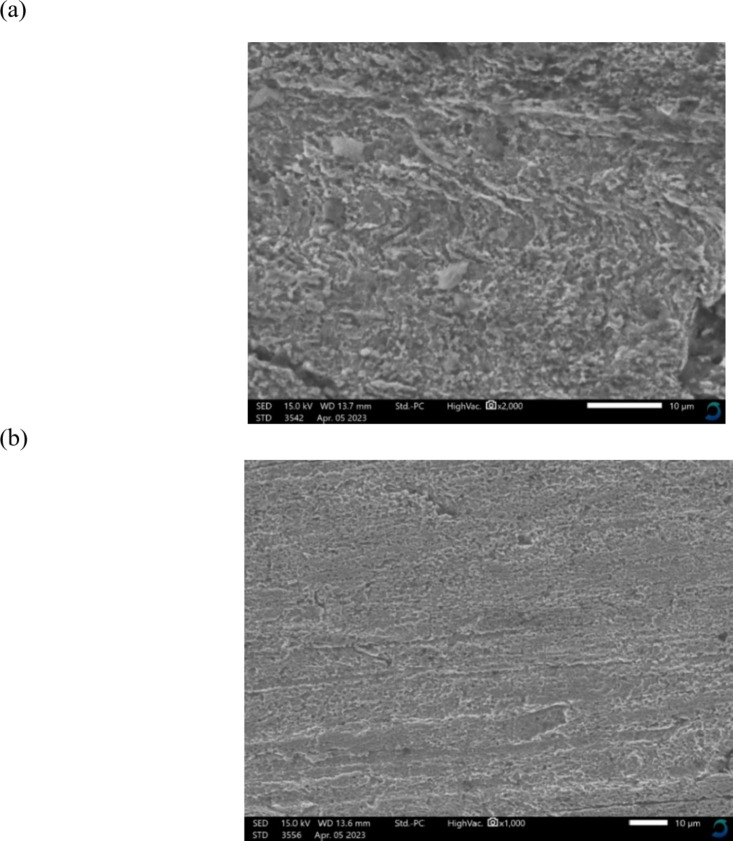
(**a**) SEM micrograph of copper surface after 24 h of inundation in 2 M HCl. (**b**) SEM micrograph of copper surface after 24 h of inundation in 2 M HCl + 123.87 × 10^− 5^ M of **GRD**.

**Fig. 6:**
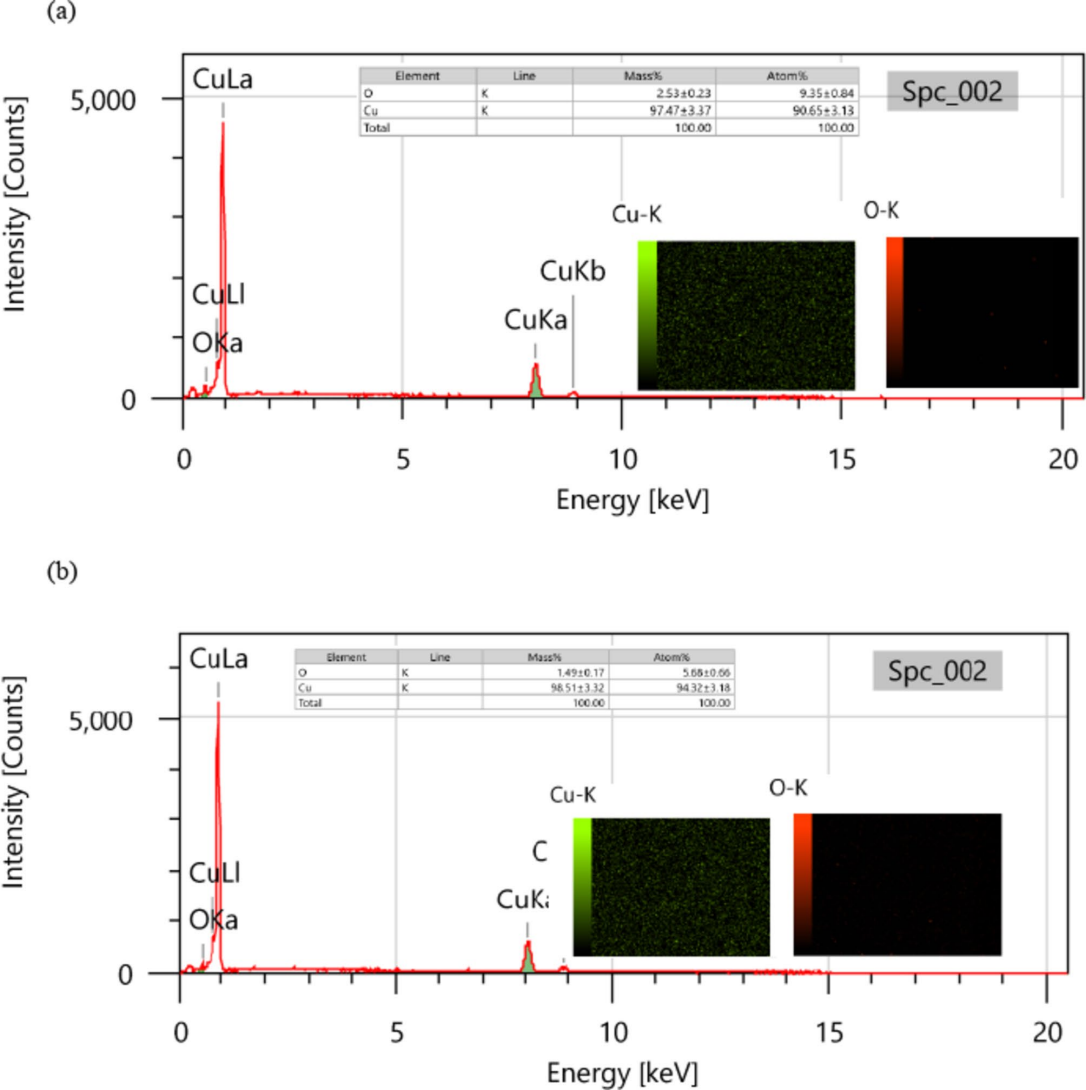
(**a**) EDX and elemental analysis of copper surface after 24 h of inundation in 2 M HCl. (**b**) EDX and elemental analysis of copper surface after 24 h of inundation in 2 M HCl + 123.87 × 10^− 5^ M of **GRD**.

Figure 5a presents a SEM picture of the copper surface following 24 h of immersion in 2 M HCl, whereas Fig. 5b displays a SEM image of a different copper specimen surface after 24 h of immersion in 2 M HCl combined with 123.87 × 10^− 5^ M of **GRD**. The micrographs indicate significant surface damage due to corrosion without the inhibitor (blank), but the presence of the inhibitor results in much reduced surface damage. This is due to the development of an effective protective layer on the copper surface. The EDX spectrum of the copper sample immersed in 2 M HCl indicated failure in the absence of inhibitor molecules due to significant external corrosion, as seen in Fig. 6a. The introduction of 123.87 × 10^− 5^ M of **GRD** inhibitor significantly enhanced the copper sample’s surface through the formation of a protective film of surfactant molecules, as evidenced by the reduction of the copper peak in Fig. 6b, indicating that the protective film was strongly adherent to the copper surface, resulting in a high level of inhibition efficiency^32,33^.

**Fig. 7:**
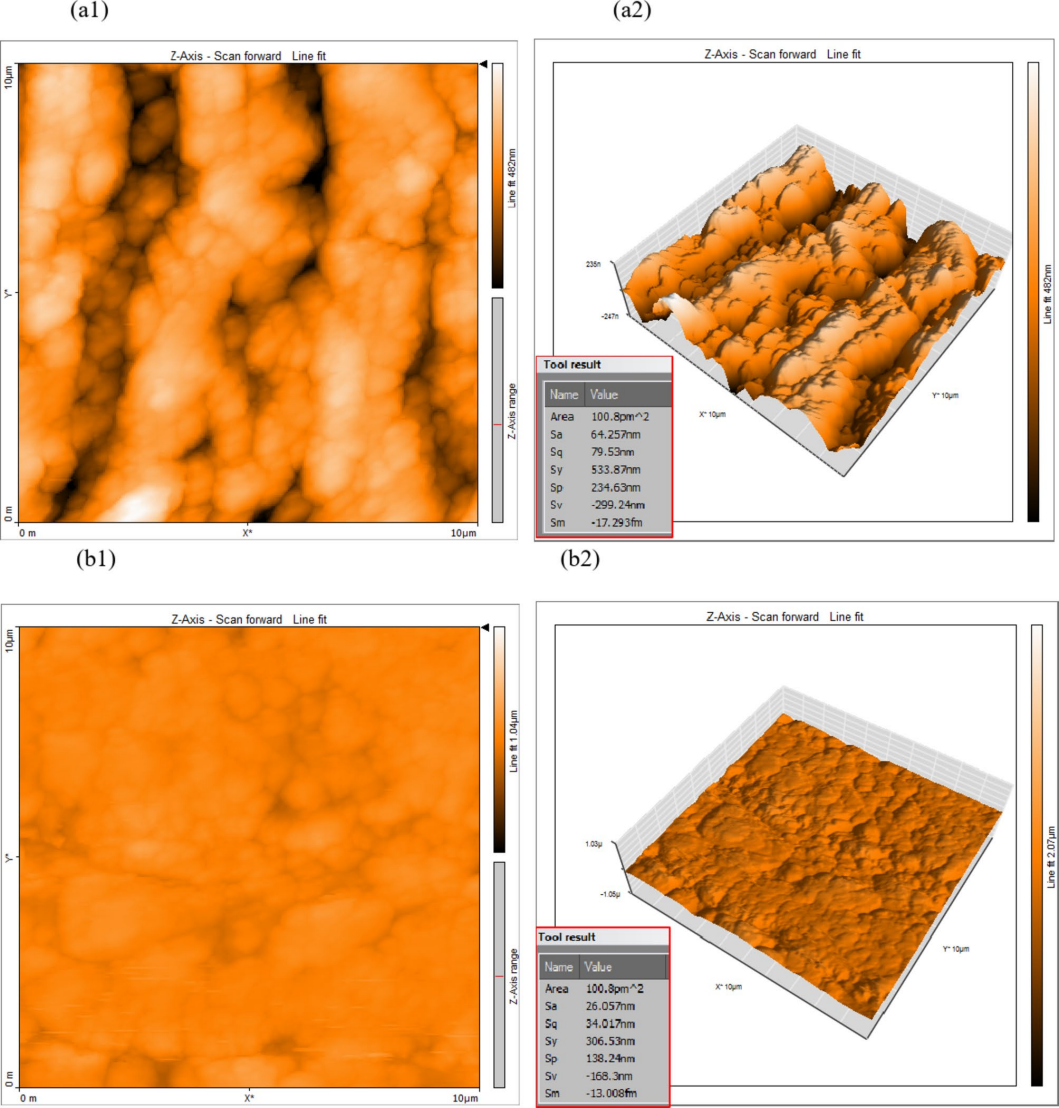
(**a1** and** a2**) 2D & 3D AFM micrographs for the free copper surface. (**b1** and** b2**) 2D & 3D AFM micrographs for copper surface after 24 h of inundation in 2 M HCl + 123.87 × 10^− 5^ M of **GRD**.

The copper surface microstructure was examined using atomic force microscopy (AFM), and further investigation into the GRD’s ability to inhibit corrosion was also done. Figure 7 shows the copper surface following corrosion testing with and without a **GRD** concentration of 123.87 × 10^− 5^ M. On the basis of Fig. 7a, the copper surface in 2 M HCl has an average roughness and root mean square of 64.25 nm and 79.53 nm, which are larger than those of a copper sample in **GRD** (Fig. 7b), which are 26.06 nm and 34.01 nm, respectively. The findings of this investigation show that GRD molecules adsorb on copper’s surface, forming a protective layer that successfully protects the metal from undesirable ions^34,35^.

### Adsorption Isotherm

**Fig. 8:**
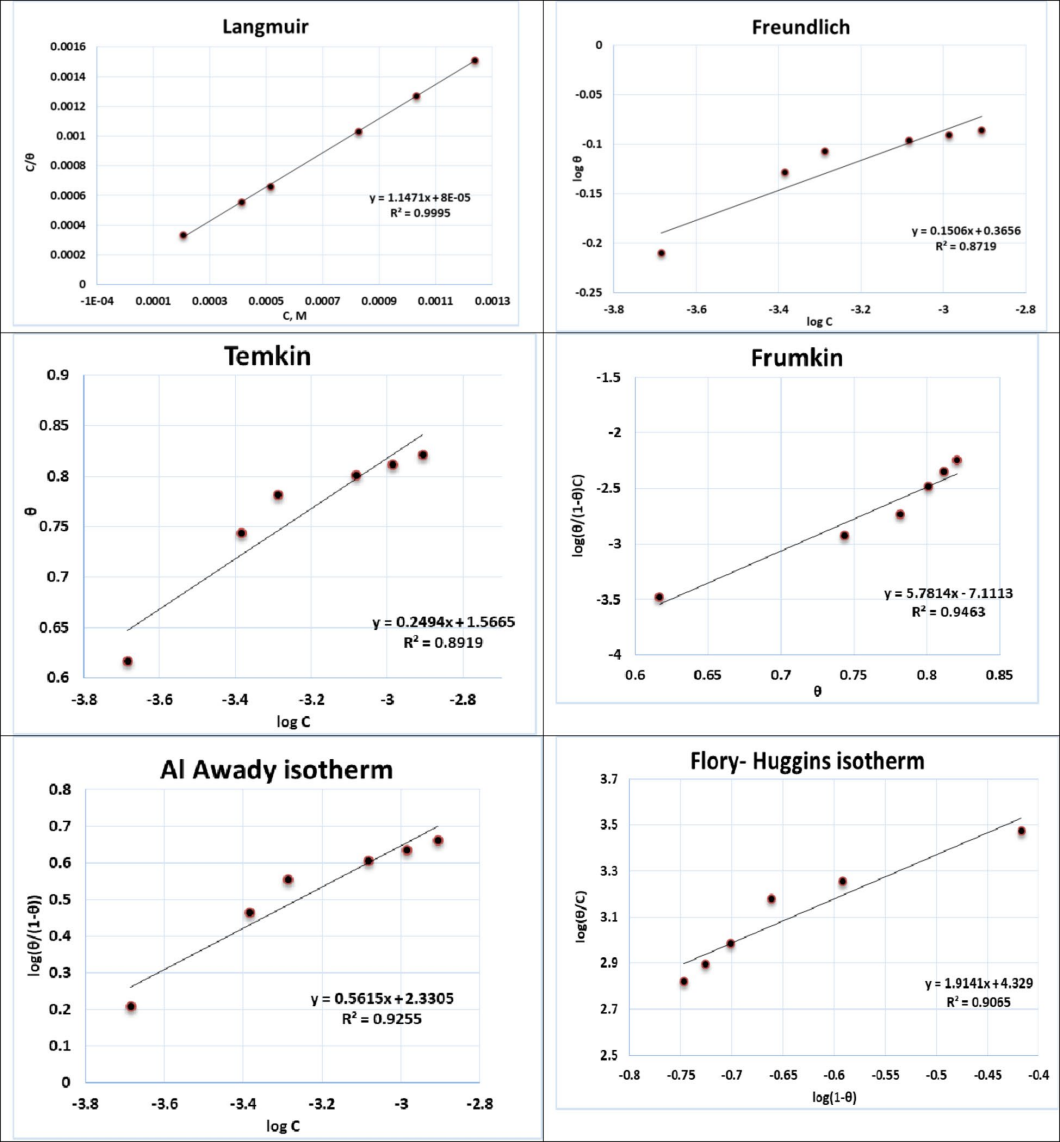
Different adsorption isotherm types for the copper electrode in 2 M HCl solution containing different concentrations of the **GRD** 25 ± 1.

Gast Reg drug (**GRD**) molecule adsorption onto metallic surfaces is the key corrosion inhibition mechanism. Two common adsorption processes, namely physisorption and chemisorption can be expected. The occurrence of physical adsorption requires the existence of metal surfaces that possess electrical charges, as well as charged species that are present within the solution’s bulk^33^. However, in chemisorption, the inhibitor molecule transfers some of its charges to the metal surface. Adsorption can be classified as physisorption, chemisorption, or a combination of both, depending on the existing forces. To clarify the adsorption mode of **GRD** molecules on the copper surface in acidic solution, the degree of surface coverage (θ) at various concentrations of **GRD** in 2 M HCl solution was determined from corresponding electrochemical polarization measurements using the formula: *θ* = ($\:\frac{icorr-icorr\:\left(inh\right)}{icorr}$). These *θ* values were fitted to different isotherms, such as Langmuir, Temkin, and Frumkin isotherms^34,35^. As represented in Fig. 8, the Langmuir adsorption isotherm, expressed as *KC* = $\:\frac{Ɵ}{(1-\:Ɵ)}$, emerged as the most suitable fitting, where *C* is the concentration of **GRD**, *θ* is the surface coverage and *K* is the equilibrium constant of the **GRD** adsorption process constant related to the free energy of adsorption *ΔG*_*ads*_ as^36^: *K* = $\:\frac{1}{Csolvent}\text{exp}\left(-\frac{\varDelta\:G}{Rt}\right)$, where *T* is the absolute temperature, *R* is the universal gas constant, and *c*_solvent_, which stands for the water concentration, is equal to 55.5 mol dm^−3^. This isotherm assumes equivalent adsorption sites and independent binding events at these sites, forming a linear relationship when *C/θ* is plotted against *C* according to *KC*= $\:\frac{\theta\:}{1-\theta\:}$, which can be rearranged to get the following formularization^23,37^: $\:\frac{C}{\theta\:}$ = $\:\frac{1}{K}$+ C. The experimentally derived equilibrium constant (*K*) is 1111.11, and the standard free energy of adsorption (*ΔG°*_*ads*_) can be calculated using the equation^7,38^: *ΔG°*_*ads*_ = -*RT* ln(55.5 *K*_*ads*_), The calculated *ΔG°*_*ads*_ value is -33.04 kJ mol^−1^, demonstrating the spontaneity of the adsorption process. It further indicates that the major mode of adsorption is mixed type as represented in Table 3^39,40^.

**Table 3:** Langmuir adsorption parameters for the copper dissolution in 2 M HCl involving different concentrations of **GRD** by polarization measurements at 25 °C:

Inhibitor	− ΔG°_ads_ (kJ mol^-1^)	K, M^-1^	Slope	*R* ^2^
GRD	33.33	12,500	1.147	0.9995

### Theoretical approaches

#### DFT studies

**Fig. 9:**
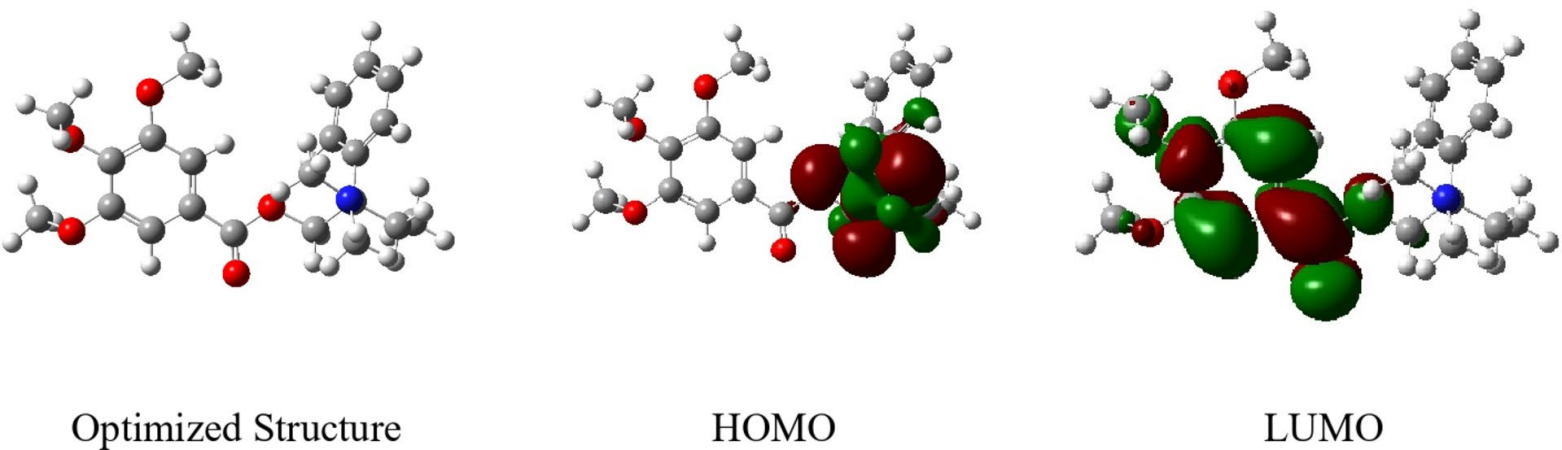
Optimized molecular structure with electronic densities of HOMO and LUMO of the tested compound **GRD**.

We examined how Gast Reg drug (**GRD**) molecules’ active parts interact with copper metal using Density Functional Theory (DFT). GRD’s optimized structures and HOMO and LUMO orbitals are shown in Fig. 9. The appropriate quantum chemical parameters are provided in Table 4. According to the Frontier Molecular Orbital (FMO) hypothesis, the HOMO and LUMO energies of an inhibitor molecule dictate whether it can donate or absorb electrons when it comes into contact with the metal surface^41^. Higher *E*_*HOMO*_ and lower *E*_*LUMO*_ values signify a highly effective corrosion inhibitor. Table 4; Fig. 9 show that the phenyl ring and amino group have the highest HOMO level, suggesting that the π-bonds and nitrogen atoms are ideal for electrophilic assaults on the copper surface. LUMO exhibits significant electronic density distributed over most of the molecules. These results back up the idea that inhibitor molecules can effectively adsorb on the surface of the copper, which is in line with the results of the studied measurements. The energy gap, denoted as *ΔE*, plays a pivotal role as an important indication, where lower values of Δ*E* are linked to increased competence in corrosion prevention shown by the inhibitor molecule^42^. Additionally, low electronegativity (*X*) values indicate increased potential reactivity of the **GRD** molecules in providing electrons to the copper surface^43^. Furthermore, the stability and reactivity of the molecule, measured through hardness (*η*) and softness (*σ*), highlight that the soft molecule, characterized by high reactivity, offers effective corrosion inhibition through smooth electron delivery to the metal surface through adsorption^44^. The dipole moment (µ) is a major indication determining corrosion inhibition^45^, where a higher dipole moment increases deformation energy and metal surface molecule adsorption. The calculated dipole moment (µ = 3.9) supports an increase in reactivity in comparison to a medium without a solvent, suggesting improved corrosion inhibition efficacy^46^.

**Table 4:** Different quantum chemistry descriptors of the tested compound GRD; global hardness (**η**), global softness **σ**(**S**), ionization potential (**I**), Electronegativity (**X**), Dipole moment (**µ**).

Quantum Parameters	Total Energy (a.u)	E_HOMO_, eV	E_LUMO_, eV	ΔE, eV	ƞ	σ(S)	I	X	µ, Debye
**GRD** / Water	− 3016.0	− 0.2061	− 0.05717	0.149	0.074	13.43	-0.132	0.132	3.9

**Fig. 10:**
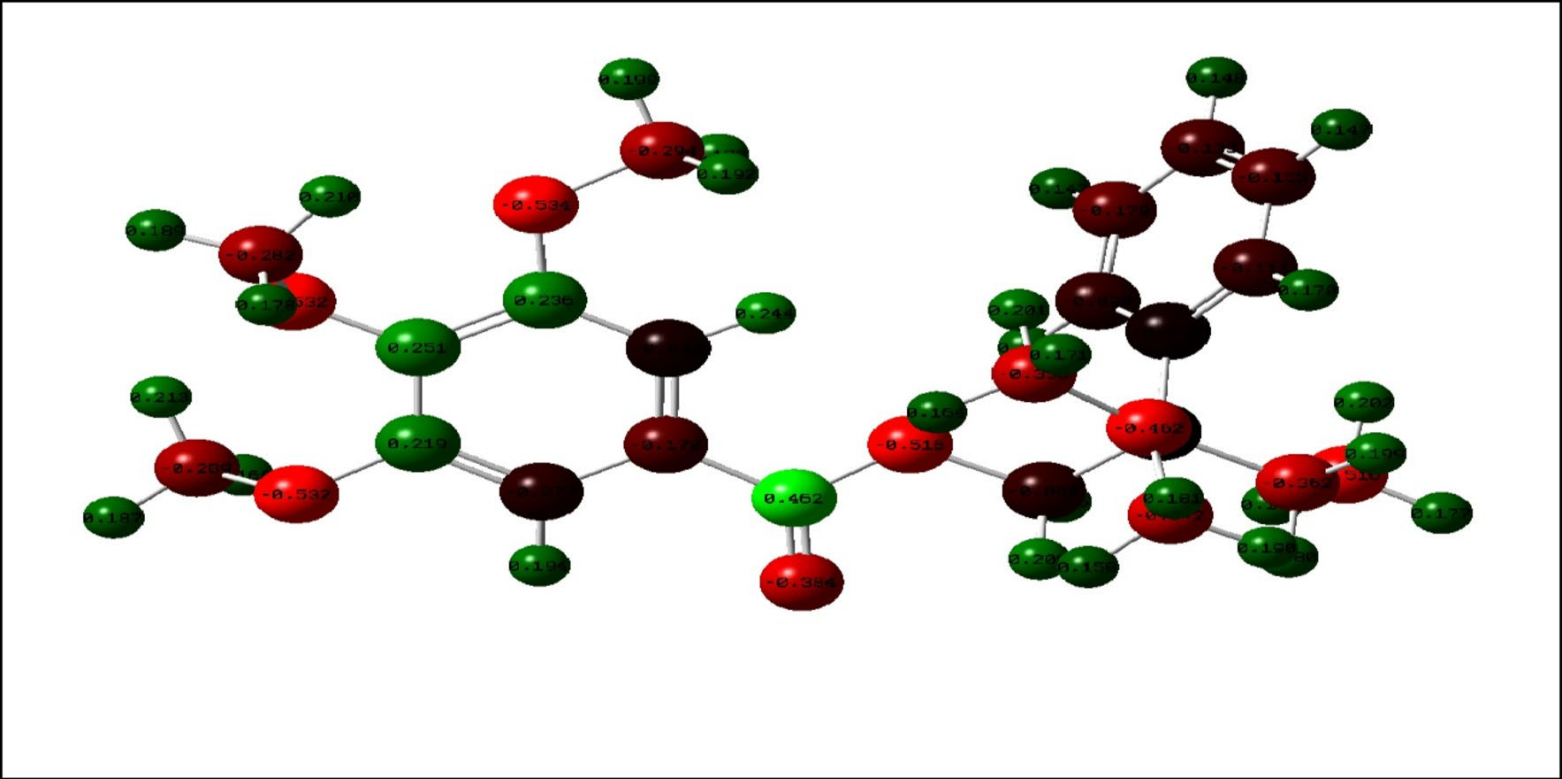
Mulliken population analysis with atomic charge ranges from -ve charged with red color and + ve charged with green color of the tested compound **GRD**.

The values of the Mulliken charge distribution and the polar direction (moment) are displayed in Fig. 10 and documented in Table 5. The high electronic intensity regions, particularly on some carbon atoms and heteroatoms (N, O), denote active cores vulnerable to electrophilic attacks^47^. According to previous research, some heteroatoms with highly negatively charged regions form a thin deposit on the metal surface via donor-acceptor-style reactions^48,49^.

**Table 5:** Mulliken charges on the atoms of the tested compound GRD.

Mulliken charges on the atoms of GRD	
Atoms	Mulliken charges
1 C	− 0.543542
2 C	− 0.139115
3 C	− 0.319130
4 C	0.073764
5 O	**− 0.515480**
6 C	0.850755
7 O	**− 0.522292**
8 C	0.648601
9 C	− 0.705436
10 C	0.315240
11 O	**− 0.436147**
12 C	− 0.130952
13 C	0.303044
14 O	**− 0.469413**
15 C	− 0.133710
16 C	0.167542
17 O	**− 0.549658**
18 C	− 0.158341
19 C	− 0.377311
20 N	**− 0.111231**
21 C	− 0.328451
22 C	− 0.176843
23 C	0.548794
24 C	− 0.304316
25 C	− 0.309637
26 C	− 0.108661
27 C	− 0.321053
28 C	− 0.153298
29 H	0.129337
30 H	0.129456
31 H	0.138364
32 H	0.141352
33 H	0.144268
34 H	0.155537
35 H	0.168267
36 H	0.119602
37 H	0.141622
38 H	0.143923
39 H	0.136141
40 H	0.140768
41 H	0.144763
42 H	0.138820
43 H	0.153765
44 H	0.155934
45 H	0.153534
46 H	0.157453
47 H	0.133332
48 H	0.145711
49 H	0.136322
50 H	0.133821
51 H	0.143976
52 H	0.125467
53 H	0.100968
54 H	0.095904
55 H	0.093431
56 H	0.094597
57 H	0.109840

Bold values indicate the hetero atoms with more negative Mulliken charges.

**Fig. 11:**
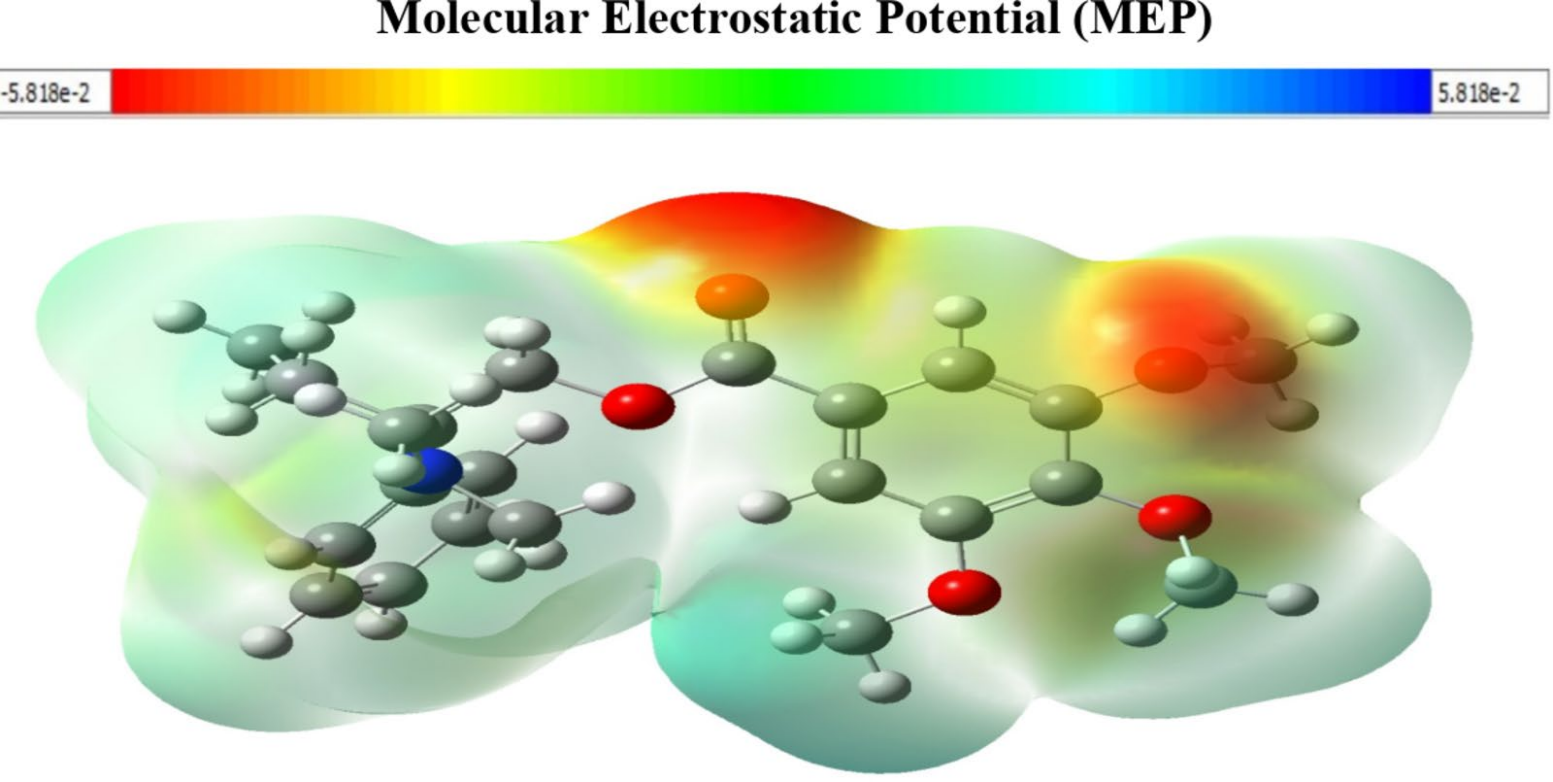
Graphical presentation of the MEP of **GRD** molecules using DMol3 module.

The Dmol^3^ module was used to perform Molecular Electrostatic Potential Mapping (MEP) for the purpose of identifying the active sites of inhibitor compounds. MEP mapping, a three-dimensional visual descriptor, shows the net electrostatic influence on a molecule by showing charge distribution^50^. As shown in Fig. 11, red colors indicate maximum electron density regions (nucleophilic reaction), with the highest negative areas primarily over carbonyl groups^51^. The presence of a lower electron density above the phenyl ring and amino group indicates the existence of potential electrophilic reaction sites. In contrast, the molecular electrostatic potential (MEP) demonstrates the highest magnitude of positive charge distribution in inhibitor compounds specifically localized around hydrogen atoms. The inhibitor molecules possess areas of heightened electron density, shown by the red parts. These locations are considered favorable for establishing contacts with the copper surface, resulting in the formation of a strongly adsorbed shielding layer.

### MC simulations

**Fig. 12:**
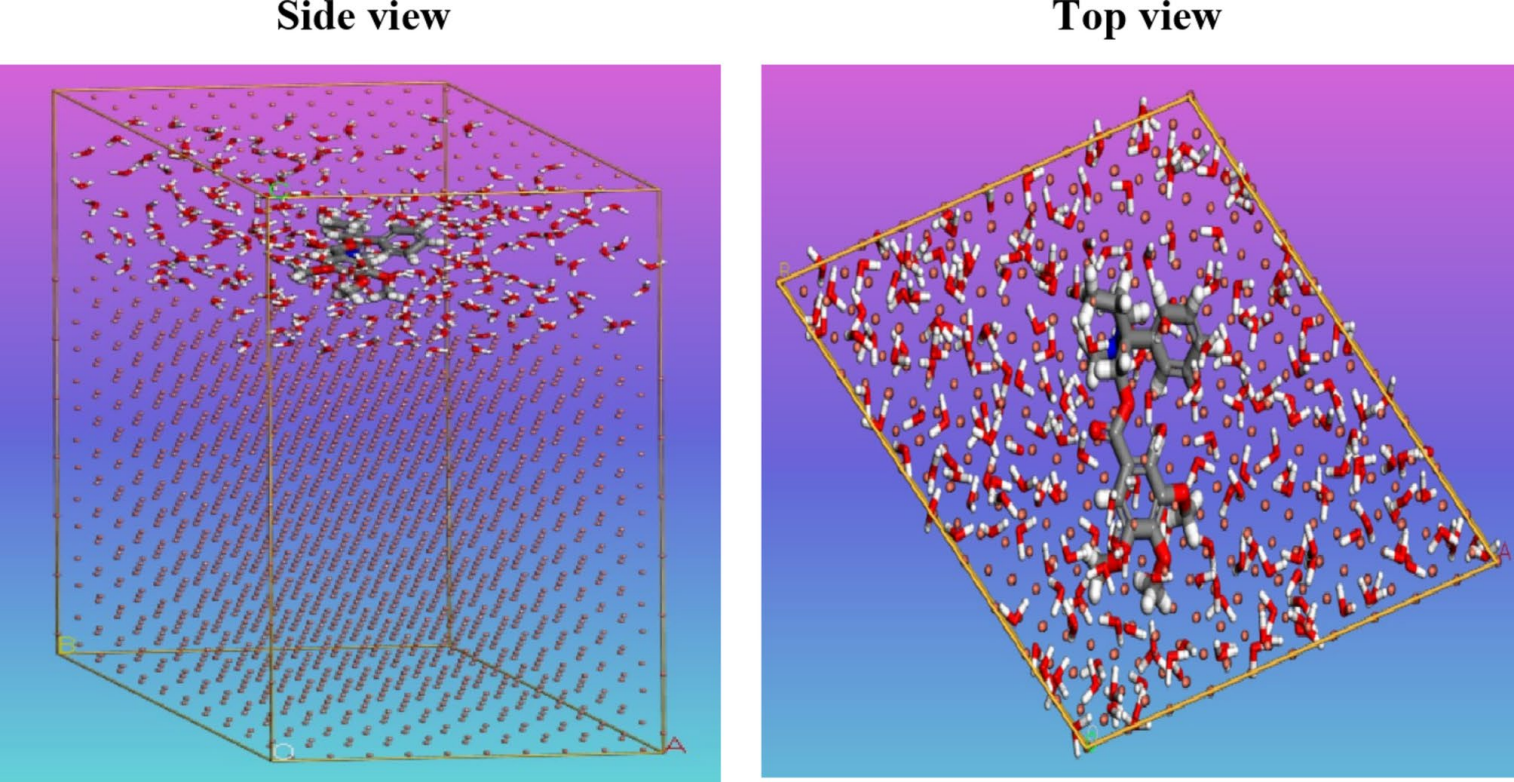
Side and top view snapshots of the most stable orientation of the tested compound **GRD** simulated as whole part in water condition.

The inhibitor molecules’ attachment to the copper metal surface and its interactions were thoroughly understood using Monte Carlo (MC) computer simulations. Figure 12 shows the adsorption locator module’s best inhibitor molecule adsorption configurations on copper. These designs’ virtually planar patterns suggest improved adsorption and maximum surface coverage^52^. The MC simulation adsorption energies parameters are shown in Table 6. Notably, **GRD** exhibits a higher negative adsorption energy value (-3371.0 kcal mol^− 1^), indicative of robust adsorption on the copper surface. This suggests the development of a strongly adsorbed layer that effectively inhibits the copper corrosion, which is consistent with the results obtained in experimental studies^53^. Adsorption energy estimates for GRD are shown in Table 6 for both the unrelaxed state (-3244.3 kcal mol^− 1^) and the relaxed state (-126.7 kcal mol^− 1^) before and after geometry adjustment, respectively. The higher inhibition proficiency for GRD is supported by these values. The dEads/dN_i_ values, representing the metal-adsorbates configuration energy when the adsorbed inhibitors or water molecules are omitted, are crucial in understanding the strength of adsorption^54^. For **GRD**, the dEa_ds_/dN_i_ value is (-122.5 kcal mol^− 1^), as presented in Table 6, which demonstrates exceptional adsorptive properties. In addition, the surrounding water has a low dE_ads_/dN_i_ ratio (-16.3 kcal mol^− 1^), indicating that inhibitor molecules are being adsorbed more strongly than water molecules. Hence, **GRD** exhibits strong adhesion to the surface of the copper, developing in the formation of a durable protective layer. This layer effectively safeguards the copper surface against corrosion when exposed to corrosive environments. Both empirical and computational analyses support this result.

**Table 6:** Data and descriptors calculated by the Monte Carlo simulation (MC) for adsorption of the tested compound **GRD** on Cu (111):

Structures	Adsorption energy/kcal mol^− 1^	Rigid adsorption energy/kcal mol^− 1^	Deformation energy/kcal mol^− 1^	dE_ads_/dN_i_: GRD kcal mol^− 1^	dE_ads_/dN_i_: Water kcal mol^− 1^
Cu (111)	− 3371.0	− 3244.3	− 126.7	− 122.5	− 16.3
**GRD**					
Water					

### The inhibition mechanism

The organic compounds that make up our inhibitor bind to metal surfaces, preventing further metal breakdown by cathodic or anodic processes, or both. Organic inhibitors have the ability to form unbreakable complexes or chelates with the metal ions present on their surface^55^. The assessment of the corrosion inhibition effectiveness of **GRD** (major constituents) in the presence of copper corrosion in a 2 M hydrochloric acid solution can be comprehended by considering various factors. These factors include the quantity of adsorption sites, the density of their charge, the size of the molecules, the way they interact with the copper surface, and their capacity to form a complex with the metallic substrate. The π-electrons and free electrons associated with the oxygen and nitrogen atoms establish chemical interactions with the copper metal surface. Based on the potential-pH diagram^56^, it has been shown that copper oxide coatings with protective properties, namely Cu_2_O and CuO, tend to readily dissolve under conditions of low pH. The following steps of actions may be used to explain how copper dissolves in an acidic environment:

Cu − e^−^→ Cu(I)_ads_ (fast).

Cu(I)_ads_ − e^−^→ Cu(II) (slow).

Cu(I)_ads_ denote a species that has been adsorbed onto the copper surface and doesn’t exhibit diffusion into the bulk solution. The process of reducing oxygen at the cathode may be represented by the following equation:

O_2_ + 4 H^+^ + 4e^−^→ 2H_2_O.

Two mechanisms have been hypothesized in the literature to explain the high inhibitory efficiency seen for the inhibitors (INH)^57^. One possible explanation for this phenomenon is the creation of a layer of INH that is adsorbed onto the surface of the metal.

Cu − e^−^→ Cu(I)_ads_ (fast).

Cu(I)_ads_ − e^−^→ Cu(II) (slow).

Cu(S) + INH → Cu∶INH_(ads)_.

where Cu: INH_(ads)_ refers to INH adsorbed on the copper surface. The other mechanism proposes the formation of a protective coating composed of Cu(I) IN on the surface, which effectively hinders the anodic dissolution process., i.e.,

Cu^+^_(aq)_ + INH_(aq)_ → Cu(I)IN_(S)_ + H^+^_(aq)_

Less acidic media prefer the Cu(I)IN complex at higher anodic potentials, whereas more acidic media favor the formation of the adsorbed species at higher cathodic potentials.

## Conclusions

This work used theoretical and empirical methods to investigate Gast Reg drug (**GRD**)’s suppression of copper corrosion during acid pickling. **GRD** demonstrated effective inhibition against copper corrosion, with its inhibitory effects increasing with higher concentrations. The maximum protection capacity reached 85.5%. Polarization investigations suggested that **GRD** operated as a mixed-type inhibitor. **GRD** adsorbed onto the metal surface, successfully preventing copper corrosion in HCl solution, according to SEM, EDX, and AFM investigations. The Langmuir isotherm model was employed to describe the adsorption behavior on the metal surface. Density Functional Theory (DFT) calculations illustrated that **GRD** interacted with the metal interface through donor–acceptor attractions. Additionally, Monte Carlo (MC) simulations suggested that **GRD** spontaneously adsorbed via its electron-rich sites.

## Data Availability

All data generated or analysed during this study are included in this published article.

## References

[1] Jones, D. A. Principles and prevention. *Corrosion* **2**, 168 (1996).

[2] Shih, H. & Tzou, R. Effect of benzotriazole on the stress corrosion cracking and the electrochemical polarization of 70/30 brass in fluoride solutions. *J. Electrochem. Soc.* **138** (4), 958 (1991).

[3] Abbas, M. Effects of temperature on dezincification and electrochemical behaviour of 70–30 brass in sulphuric acid. *Br. Corros. J.* **26** (4), 273–278 (1991).

[4] Al-Matrouk, F. et al. *Industrial Corrosion and Corrosion Control Technology*, 567–579 (Kuwait Institute for Scientific Research, 1996).

[5] Quartarone, G. et al. Using indole to inhibit copper corrosion in aerated 0.5 M sulfuric acid. *Corrosion* **54** (8), 606–618 (1998).

[6] Sobhi, M. & Eid, S. Chemical, electrochemical and morphology studies on methyl hydroxyethyl cellulose as green inhibitor for corrosion of copper in hydrochloric acid solutions. *Prot. Met. Phys. Chem. Surf.* **54**, 893–898 (2018).

[7] Eid, S. et al. Surface, electrochemical, and theoretical investigation on utilizing olive leaf extract as green inhibitor for copper corrosion in alkaline environment. *Arab. J. Sci. Eng.* **49** (1), 147–164 (2024).

[8] Althobaiti, I. O. et al. Evaluation of the impact of two thiadiazole derivatives on the dissolution behavior of mild steel in acidic environments. *Molecules* **28** (9), 3872 (2023). 37175282 10.3390/molecules28093872 PMC10180302

[9] Attia, M. et al. Experimental and theoretical study on some azo chromotropic acid dyes compounds as inhibitor for carbon steel corrosion in sulfuric acid. *J. Iran. Chem. Soc.* **19** (2), 655–664 (2022).

[10] Eid, S. Expired Desloratidine drug as inhibitor for corrosion of carbon steel pipeline in hydrochloric acid solution. *Int. J. Electrochem. Sci.* **16** (1), 150852 (2021).

[11] Fouda, A. et al. Tenormin drug as save corrosion inhibitor for 304 stainless steel in hydrochloric acid solutions. *Der Pharma Chem.* **7** (4), 22–33 (2015).

[12] Khudhair, N. A., Kadhim, M. M. & Khadom, A. A. Effect of trimethoprim drug dose on corrosion behavior of stainless steel in simulated human body environment: experimental and theoretical investigations. *J. Bio Tribo-Corros*. **7** (3), 124 (2021).

[13] Ganapathi Sundaram, R., Vengatesh, G. & Sundaravadivelu, M. Adsorption behavior and anticorrosion capability of antibiotic drug nitroxoline on copper in nitric acid medium. *J. Bio Tribo-Corros*. **3**, 1–13 (2017).

[14] Maduelosi, N. J. & Iroha, N. B. Insight into the adsorption and inhibitive effect of spironolactone drug on C38 carbon steel corrosion in hydrochloric acid environment. *J. Bio Tribo-Corros.* **7** (1), 6 (2021).

[15] Eddy, N. O., Ebenso, E. E. & Ibok, U. J. Adsorption, synergistic inhibitive effect and quantum chemical studies of ampicillin (AMP) and halides for the corrosion of mild steel in H 2 SO 4. *J. Appl. Electrochem.* **40**, 445–456 (2010).

[16] Geethamani, P. et al. Corrosion inhibition and adsorption properties of mild steel in 1 M hydrochloric acid medium by expired ambroxol drug. *J. Bio Tribo-Corros.* **5**, 1–18 (2019).

[17] El-Katori, E. E. & Abousalem, A. S. Impact of some pyrrolidinium ionic liquids on copper dissolution behavior in acidic environment: experimental, morphological and theoretical insights. *RSC Adv.* **9** (36), 20760–20777 (2019). 35515546 10.1039/c9ra03603b PMC9065807

[18] El-Katori, E. E., Ahmed, M. & Nady, H. Imidazole derivatives based on glycourils as efficient anti-corrosion inhibitors for copper in HNO3 solution: synthesis, electrochemical, surface, and theoretical approaches. *Colloids Surf. A Physicochem. Eng. Aspects* **649**, 129391 (2022).

[19] Farahati, R. et al. Evaluation of corrosion inhibition of 4-(pyridin-3-yl) thiazol-2-amine for copper in HCl by experimental and theoretical studies. *J. Mol. Struct.* **1205**, 127658 (2020).

[20] Badawy, W. A., Ismail, K. M. & Fathi, A. M. Corrosion control of Cu–Ni alloys in neutral chloride solutions by amino acids. *Electrochim. Acta* **51** (20), 4182–4189 (2006).

[21] Radovanović, M. B. et al. Electrochemical and DFT studies of brass corrosion inhibition in 3% NaCl in the presence of environmentally friendly compounds. *Sci. Rep.* **9** (1), 16081 (2019). 31695132 10.1038/s41598-019-52635-2 PMC6834559

[22] Ismail, K. M. Evaluation of cysteine as environmentally friendly corrosion inhibitor for copper in neutral and acidic chloride solutions. *Electrochim. Acta* **52** (28), 7811–7819 (2007).

[23] Syam, S. et al. An examination of the effectiveness of the expired drug isoprinosine in preventing aluminum corrosion in alkaline solutions using both computational and experimental techniques. *RSC Adv.* **14** (16), 11244–11257 (2024). 38590354 10.1039/d4ra00158c PMC11000097

[24] Abd El-Hafez, G. M. & Badawy, W. A. The use of cysteine, N-acetyl cysteine and methionine as environmentally friendly corrosion inhibitors for Cu–10Al–5Ni alloy in neutral chloride solutions. *Electrochim. Acta* **108**, 860–866 (2013).

[25] Salah Eid, K. A., Soliman, A. Y., El–Etre, E., Gad & Nady, H. Combination of experimental and computational insight into the anti-corrosion performance of 1-(4-tert-butylphenyl)-4-(4-(benzhydryloxy)piperidin-1-yl)butan-1-one onto C-steel in acidic environments.* J. Bio Tribo-Corrosion* **9**(61), 1–14 (2023).

[26] Khadom, A. A. & Yaro, A. S. Mass transfer effect on corrosion inhibition process of copper–nickel alloy in hydrochloric acid by Benzotriazole. *J. Saudi Chem. Soc.* **18** (3), 214–219 (2014).

[27] Macdonald, J. *WB Johnson in: JR Macdonald. Impedance Spectroscopy*, 150–170 (Wiley, 1987).

[28] Mansfeld, F. & Shih, H. Detection of pitting with electrochemical impedance spectroscopy. *J. Electrochem. Soc.* **135**, 1171–1172 (1988).

[29] Badawy, W., El-Rabiei, M. & Nady, H. Synergistic effects of alloying elements in Cu-ternary alloys in chloride solutions. *Electrochim. Acta* **120**, 39–45 (2014).

[30] Ma, H. et al. Inhibition of copper corrosion by several Schiff bases in aerated halide solutions. *J. Appl. Electrochem.* **32**, 65–72 (2002).

[31] Sherif, E. & Park, S. M. Inhibition of copper corrosion in acidic pickling solutions by N-phenyl-1, 4-phenylenediamine. *Electrochim. Acta* **51** (22), 4665–4673 (2006).

[32] Chaubey, N. et al. A comparative study of leaves extracts for corrosion inhibition effect on aluminium alloy in alkaline medium. *Ain Shams Eng. J.* **8** (4), 673–682 (2017).

[33] Quartarone, G. et al. Investigation of the inhibition effect of indole-3-carboxylic acid on the copper corrosion in 0.5 M H2SO4. *Corros. Sci.* **50** (12), 3467–3474 (2008).

[34] Khaled, K., Fadl-Allah, S. A. & Hammouti, B. Some benzotriazole derivatives as corrosion inhibitors for copper in acidic medium: experimental and quantum chemical molecular dynamics approach. *Mater. Chem. Phys.* **117** (1), 148–155 (2009).

[35] Quartarone, G. et al. Inhibitive action of gramine towards corrosion of mild steel in deaerated 1.0 M hydrochloric acid solutions. *Corros. Sci.* **64**, 82–89 (2012).

[36] Ross, P. N. *Adsorption of molecules at metal electrodes* (1992).

[37] Abdallah, M. et al. Animal glue as green inhibitor for corrosion of aluminum and aluminum-silicon alloys in sodium hydroxide solutions. *J. Mol. Liq.* **220**, 755–761 (2016).

[38] Abdallah, M. et al. Natural occurring substances as corrosion inhibitors for tin insodium bicarbonate solutions. *J. Korean Chem. Soc.* **53** (5), 485–490 (2009).

[39] Syam, S. et al. Combination of practical and theoretical measurements of albumin egg as an eco-friendly inhibitor for copper corrosion in alkaline solutions. *RSC Adv.* **13** (48), 33929–33942 (2023). 38020017 10.1039/d3ra05835b PMC10658222

[40] Solmaz, R., Altunbaş, E. & Kardaş, G. *Adsorption and corrosion inhibition effect of 2-((5-mercapto-1, 3, 4-thiadiazol-2-ylimino) methyl) phenol Schiff base on mild steel.** Mater. Chem. Phys.*** 125**(3), 796–801 (2011).

[41] Boulhaoua, M. et al. Synthesis, structural analysis and corrosion inhibition application of a new indazole derivative on mild steel surface in acidic media complemented with DFT and MD studies. *Colloids Surf. A Physicochem. Eng. Aspects* **617**, 126373 (2021).

[42] Abd El-Lateef, H. M., Shalabi, K. & Tantawy, A. H. Corrosion inhibition and adsorption features of novel bioactive cationic surfactants bearing benzenesulphonamide on C1018-steel under sweet conditions: combined modeling and experimental approaches. *J. Mol. Liq.* **320**, 114564 (2020).

[43] Palaniappan, N., Cole, I. & Kuznetsov, A. Experimental and computational studies of graphene oxide covalently functionalized by octylamine: Electrochemical stability, hydrogen evolution, and corrosion inhibition of the AZ13 mg alloy in 3.5% NaCl. *RSC Adv.* **10** (19), 11426–11434 (2020). 35495345 10.1039/c9ra10702a PMC9050467

[44] Obot, I., Macdonald, D. & Gasem, Z. Density functional theory (DFT) as a powerful tool for designing new organic corrosion inhibitors. Part 1: an overview. *Corros. Sci.* **99**, 1–30 (2015).

[45] Lukovits, I., Kalman, E. & Zucchi, F. Corrosion inhibitors—correlation between electronic structure and efficiency. *Corrosion* **57**(1), 3–8 (2001).

[46] Upadhyay, A. et al. Verification of corrosion inhibition of mild steel by some 4-Aminoantipyrine-based Schiff bases–impact of adsorbate substituent and cross-conjugation. *J. Mol. Liq.* **333**, 115960 (2021).

[47] Abd El-Lateef, H. M., Shalabi, K. & Tantawy, A. H. Corrosion inhibition of carbon steel in hydrochloric acid solution using newly synthesized urea-based cationic fluorosurfactants: experimental and computational investigations. *New J. Chem.* **44** (41), 17791–17814 (2020).

[48] Oyebamiji, A. & Adeleke, B. Quantum chemical studies on inhibition activities of 2, 3-dihydroxypropyl-sulfanyl derivative on carbon steel in acidic media. *Int. J. Corros. Scale Inhib.* **7** (4), 498–508 (2018).

[49] Madkour, L. H., Kaya, S. & Obot, I. B. Computational, Monte Carlo simulation and experimental studies of some arylazotriazoles (AATR) and their copper complexes in corrosion inhibition process. *J. Mol. Liq.* **260**, 351–374 (2018).

[50] Gece, G. & Bilgiç, S. Quantum chemical study of some cyclic nitrogen compounds as corrosion inhibitors of steel in NaCl media. *Corros. Sci.* **51** (8), 1876–1878 (2009).

[51] Shalabi, K. et al. New pyridinium bromide mono-cationic surfactant as corrosion inhibitor for carbon steel during chemical cleaning: experimental and theoretical studies. *J. Mol. Liq.* **293**, 111480 (2019).

[52] El Aadad, H. et al. Improvement of the corrosion resistance of mild steel in sulfuric acid by new organic-inorganic hybrids of Benzimidazole-Pyrophosphate: facile synthesis, characterization, experimental and theoretical calculations (DFT and MC). *Surf. Interfaces*. **24**, 101084 (2021).

[53] Dehghani, A. et al. A detailed study on the synergistic corrosion inhibition impact of the quercetin molecules and trivalent europium salt on mild steel; electrochemical/surface studies, DFT modeling, and MC/MD computer simulation. *J. Mol. Liq.* **316**, 113914 (2020).

[54] Haque, J. et al. Pyrimidine derivatives as novel acidizing corrosion inhibitors for N80 steel useful for petroleum industry: a combined experimental and theoretical approach. *J. Ind. Eng. Chem.* **49**, 176–188 (2017).

[55] Mistry, B. et al. Experimental and quantum chemical studies on corrosion inhibition performance of quinoline derivatives for MS in 1 N HCl. *Bull. Mater. Sci.* **35**, 459–469 (2012).

[56] Mzioud, K. et al. Inhibition of copper corrosion by the essential oil of Allium sativum in 0.5 MH 2 SO 4 solutions. *SN Appl. Sci.* **2**, 1–13 (2020).

[57] Khaled, K. & Amin, M. A. Dry and wet lab studies for some benzotriazole derivatives as possible corrosion inhibitors for copper in 1.0 M HNO_*3*_. *Corros. Sci.* **51** (9), 2098–2106 (2009).

